# Does e-commerce really matter on international trade of Asian countries: Evidence from panel data

**DOI:** 10.1371/journal.pone.0284503

**Published:** 2023-04-24

**Authors:** Praveen Shanmugalingam, Ahashraaj Shanmuganeshan, Abinaya Manorajan, Mathusany Kugathasan, Geethma Yahani Pathirana

**Affiliations:** 1 SLIIT Business School, Sri Lanka Institute of Information Technology, Malabe, Sri Lanka; 2 Department of Business Management, SLIIT Business School, Sri Lanka Institute of Information Technology, Malabe, Sri Lanka; Zhejiang University, CHINA

## Abstract

Over the decades, technology has become an essential indicator to actively participate in the economic growth of nations. The usage of technology and e-commerce had created a new pathway to improve trade in Asian countries. This study seeks to verify the linkage between e-commerce and international trade. The annual data for panel data regression analysis were collected from the World Bank covering 38 Asian countries for 11 years, from 2010 to 2020. This study applied a set of estimation procedures such as descriptive statistic, correlation matrix, stationary test (Levin–Lin–Chu test, Breitung test, Augmented Dickey Fuller test, Harris–Tzavalis and Im–Pesaran–Shin test), Kao cointegration test, autocorrelation test and heteroskedasticity test. The two-step system Generalized Method of Moments (GMM) estimator was employed for dynamic panel data analysis. Empirical findings show that e-commerce significantly impacts the international trade of Asian countries. Governments of Asian countries should employ policies related to telecommunication technologies for e-commerce improvement and realize/ reap potential benefits from international trade.

## Introduction

In the current age, technology has a significant role in the acquisition of goods that easily transform people’s lifestyles. E-commerce is an integral part/ a key component of the world economy. The rapid increase in e-commerce expansion is due to technological advancements keeping pace with the changing face of international trade. Nowadays, e-markets are widely open and accessible to all, enabling people to exchange goods through the internet with advancements in electronic banking and logistics services. Much excitement is evident about e-commerce in wealthy nations /in leading economic powers like China and the United States. Internet’s effective involvement is palpable in this trend, where international trade goes hand in hand with e-commerce. Thus, global trade is highly connected to e-commerce. The influence of e-commerce has led to easy and straightforward transactions, which increased / enhanced the efficiency and effectiveness of international trade; consequently, the goal of this study is to determine the extent of association between e-commerce and trade. Freund and Weinhold [1] discovered the impact of the internet on international trade through a theoretical model. Lapatinas [2] stated internet had a positive impact on the economic sophistication of exports. Globalization and the evolution of the internet enable global trade to be more accessible and expand the global level relationships between buyers and sellers. Similarly, Choi and Yi [3] depicted that the internet relatively stimulates economic growth in developing nations.

Compared to studies focusing on the link between e-commerce or the internet and international trade, currently, fewer literatures are specifically devoted to this topic. Additionally, the majority of research on this topic has been conducted internationally. As Asia is the largest continent globally and considering its significance in global trade, this region is the subject of this research. In addition to past literature, as per the authors’ knowledge, there is a lack of studies incorporating the influential factors, including secure internet server (SERVER) as the Information and Communication Technology (ICT) indicator. Therefore, this study contributes to the literature by including SERVER as the independent variable available in the World Bank database during the period under study (2010–2020). Additionally, this study uses panel regression and secondary data from 2010 to 2020 to assess the effect of e-commerce on international trade in Asian nations.

This paper/ study consists of the following sections / This paper is structured as follows. The first section is the introduction, i.e. the outline of our study. The second section is the literature review, a discussion of underlying concepts and variables concerning the study; the third section describes the methodology used to examine the objective of this study. The fourth section elaborates on the results along with the discussion in the fifth section, indicating how the study objectives have been met. The sixth section marks the conclusion, including the recommendation of this study.

## Literature review

The 21^st^ century is the era of globalization, where e-commerce and technology, rapidly grew and developed globally/worldwide. E-commerce is unique and its birth in 1979 brought massive and many opportunities to businesses and countries globally/worldwide. As a new strategy or model of e-commerce, it not only gives opportunities for businesses but also has a sizable impact on international trade. E-commerce usage can bring opportunities to countries by expanding the business entities of nations through adopting the internet to minimize operational costs and compete with a wide range of global markets. Hence, a study of e-commerce on trade has practical importance in developing and boosting international trade. Yin and Choi [4] discovered that e-commerce is a kind of tech which can increase/improve productivity in many ways, simultaneously, which can be used as a tool to lower the cost.

The expansion of e-commerce follows the path of a digital environment. This progress should be identified and secured by making further considerations that must be made when using e-commerce. In addition, United Nations [5] revealed e-commerce as a significant development that has been widely hailed, which also has made a standard shift in how businesses are conducted around the world/worldwide. To support this theory, Terzi [6] found that e-commerce is simply about selling and purchasing goods and services among homes, individuals, governments, and organizations. It is a broad phrase that refers to various commercial operations. When describing profoundly, e-commerce is a new platform which creates job opportunities in line with labor market demands. Similarly, Ueasangkomsate [7] highlighted it as a gateway for small and medium-scale organizations to expand their business and seize the opportunity /tap the opportunity to attract shareholders and customers. Zhang [8] revealed that e-commerce is a platform that enables people to buy goods and services for a reasonable price, meaning that the intermediary costs can be eliminated. Goods are reasonably priced for both the suppliers and customers and with better accessibility to markets. This also signified the possibility for buyers and sellers in terms of accessibility for products/services. Shim et al. [9] further revealed that it is not only a system that includes transactions of goods and services but also creates revenue and demand for the services and goods, limiting physical barriers to a greater extent. To put it another way/From a different perspective, Bidgoli [10] highlighted e-commerce as a marketing strategy for goods and services, as well as online fund transfers. Haji [11] discovered that e-commerce is a digital economy that transfers every society and humanity to the internet. The study of Anvari and Norouzi [12] indicated that the internet is significantly expanding the potential of e-commerce through business-to-business (B2B) transactions and B2B markets. For business-to-consumer (B2C) transactions, the internet provides a feasible and fast-track path for world economic development. It provides the opportunity for the suppliers to make a transaction through virtual/online platforms with the foreign sellers where the customers’ physical presence is not needed, unlike in a conventional setting (i.e. they do not need to travel to the seller’s nations).

E-commerce promotes cross-border sales and helps reach and expand international trade. Kituyi [13] discovered that a portion of USD 1.45 billion online customers increased cross-border transactions from 17–23 percent from 2016 to 2018. Moreover, e-commerce sales were USD 25.6 trillion in 2018, a 8 percentage increase from 2017. The Internet plays a main role in activating e-commerce and seems like a primary factor in the e-commerce business. Internet and e-commerce application helps buyers to purchase products and services from suppliers without physical proximity. This way, the internet promotes trade and removes other trade barriers, especially those in a physical / conventional setting. Therefore, this study also refers to the existing literature on the internet and international trade-related studies.

This subject is supported by ample empirical evidence. Gnangnon and Iyer [14] used an unbalanced panel data set with data consisting of 175 countries from 2000 to 2013 to find the impact of internet usage on global trade among nations. The findings revealed that the increasing internet usage in countries assists them in gaining global market access. Wang and Choi [15] used the gravity model from 2000 to 2016 and analyzed the relationship between ICT and exports. The results showed that ICT has a greater positive impact on export rather than import and the effects of ICT level on trade increased over time. Their study findings indicate that if BRICS countries (Brazil, Russia, India, and China) increase the fixed broadband and internet infrastructure, in turn, will increase export volumes. Using a comparative analysis, Myovella et al. [16] investigated the linkage between digitalization and economic development in a group of countries by comparing Sub-Saharan African (SSA) countries and the Organization for Economic Cooperation and Development (OECD) nations. Here, the scholars employed a dataset consisting of 33 OECD countries and 41 SSA countries as sample countries from 2006 to 2016. Here, the scholars concluded that digitalization provides potential benefaction for the developing economies of both categories of countries. Compared to OECD countries, SSA countries have a massive effect on mobile technology for economic development. However, due to the digital divide, SSA countries show inadequate development on internet usage and broadband.

To identify the effect of trade openness, the internet, and financial expansion towards economic development in South African countries, Salahuddin and Gow [17] employed the granger causality test to measure the long-run connection between variables. Using time series data from 1991 to 2012, they concluded that a long-term connection exists among internet usage, economic development and financial growth. Using the GMM estimator, Asongu and Nwachukwu [18] examined that entrepreneurship in SSA has improved by ICT with the contingent of openness. The findings of the study suggested policies associated with ICT penetration must be implemented and put in place to develop entrepreneurship with consideration of population growth. Bojnec and Ferto [19] investigated the impact of internet subscribers on the OECD nations’ growth of industrial export. The findings explored the positive impact of internet subscribers on the growth of industrial export between the OECD nations, using the panel data regression from 1995 to 2003. Higher internet usage improves information and boosts competitive and manufacturing costs. Furthermore, internet users helped to develop the efficiency of e-commerce and e-business. Although, it implies that e-commerce application is used not only by individuals but also by organizations, which is valid with the growth of e-commerce and global trade.

Dunt and Harper [20] discovered the perspective of e-commerce and internet usage on the Australian economy. This again validates that the internet and e-commerce boost the expansion of exports. Also, both helped small and medium-sized businesses to participate in international trade. The survey shows that 69 percent of exporters used the internet from 1997 to 1998 in Australia. In addition, the internet and e-commerce gained productivity and economic growth. As such, Australia is widely considered to be convenient to benefit from e-commerce and internet usage, specially through simplified procedures and the adoption of digital technologies. Their digital trade strategy is an enabler of the digital economy by improving the information flow and access to market intelligence in addition to other opportunities brought about by digital trade.

Using the gravity model from 2013 to 2015, Xing [21] explored the effect of e-commerce adoption and the internet on trade exchange in 21 economically developing nations, less developed nations and 30 OECD nations. Moreover, findings indicated that e-commerce and ICT increased global trade/ international trade. In addition, the number of internet users, B2B and B2C markets/transactions, internet adoption, the available number of secured internet servers and broadband subscriptions have a substantial impact on bilateral commerce flows in the OECD nations. However, if the bilateral trade exchange is reversed, a significant association between ICT and trade exchange cannot be observed. Furthermore, using panel data analysis from 2000 to 2018. He et al. [22] discovered the impacts of cross-border e-commerce and global logistics in OECD nations. Moreover, findings revealed that the gross domestic product per capita (GDP per capita) is a main factor and shows the dynamic link between e-commerce and global logistics. OECD countries develop the manufacturing sector to increase the GDP per capita. This suggests that the OECD nations should pursue various associated measures, including tariff reduction and logistics formation improvements, to encourage the long-term improvement of international logistics.

Furthermore, from a different perspective/standpoint, Toader et al. [23] focused on ICT systems’ influence on European nations’ economic development. From 2000 to 2017, panel data estimations were done using a sample of 28 countries. Here, a massive positive impact of ICT infrastructure on economic growth was evident in European countries. Microeconomic factors such as foreign direct investments, unemployment rate, trade openness, inflation rate, government expenditures and ICT infrastructure are key factors of economic growth.

Barbero and Rodriguez-Crespo [24] discovered the impact of broadband subscriptions on trade in the European region, where ICT played a pivotal role in European trade. The gravity model equation was developed using panel data analysis in the sample of 232 European regions during 2007–2010. Moreover, the results depicted a positive influence of broadband on trade. The degree to which broadband is crucial for the exporter and importer regions varies based on the specific type of spatial dependence being analyzed. Ozcan [25] found out the effect of ICT on overseas trade among trading partners and the Turkish economy. A panel data analysis using a gravity model equation from 2000 to 2014 indicated that ICT affects Turkey’s exports and imports. The impact of ICT is larger in the import sector in comparison to the exports, considering a sample of 35 import countries and 34 export countries.

Using panel data from 2000 to 2018, Kurniawati [26] discovered that internet penetration enhances the economic growth of Asian countries; he specified that telephone and mobile phone penetration contains capabilities to develop the economic development of Middle-income Asian countries. Since most Middle East countries form a large part of the Asian continent, the study of Bahrini and Qaffas [27] investigated the ICT’s impact on economic growth by employing the two-step GMM estimator using data from 2007 to 2016. This study concluded that internet usage, mobile phones and broadband adoption significantly influence economic growth in the Middle East, SSA and North America. According to most studies, internet penetration positively differentiates the export margins by bridging the linguistic gap in trade, especially in the form of broadband connections [28].

**Fig 1:**
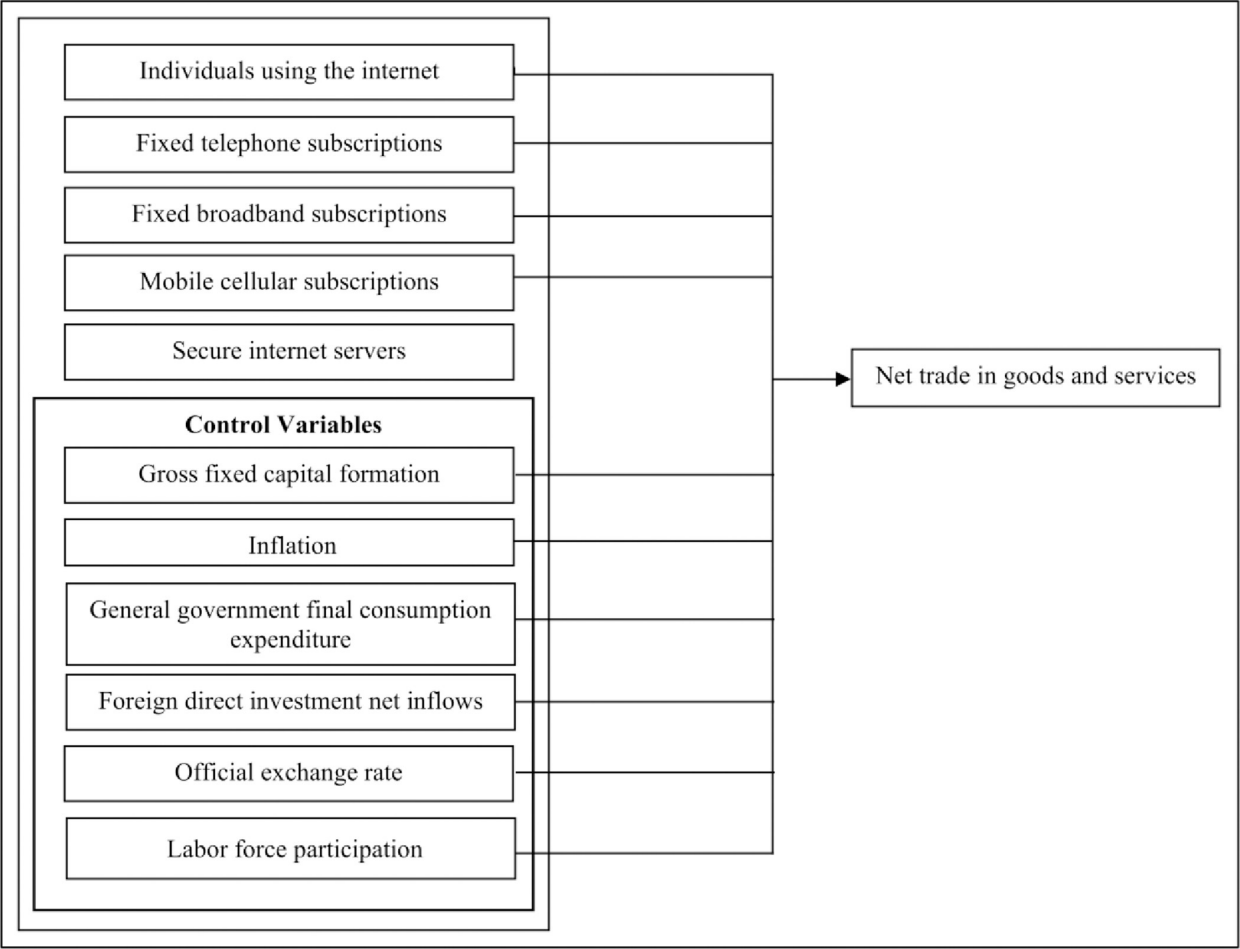
Conceptual framework. Source: Authors’ illustration.

Previous studies indicate that the internet has a significant effect on promoting international trade. Most research studies based on e-commerce and international trade were dedicated to, particularly global level or a group of countries, such as OECD, the Middle East, North Africa and developing countries. Nevertheless, some limited research studies conducted in Asia were mostly based on ‘Belt and Road Initiative’ (BRI) countries and in countries representing the Association of Southeast Asian Nations (ASEAN). Also, according to previous studies, every continent resembles unique and different representations of e-commerce facilities towards international trade. Thereby variables affecting this relationship and the magnitude of the impact too vary. America, Europe and Oceania continent have the latest ICT infrastructure in place to promote international trade, while Africa has a digital divide and mobile commerce significantly affect to enhance e-commerce. Moreover, limited studies were based in the Asian continent on these subject areas and are yet to unveil valuable discoveries in this subject through empirical research. Therefore, this study is expected to address this research gap using dynamic panel data analysis. Fig 1 represents variables which were developed based on past literature.

## Materials and methods

The objective of this study is to identify the impact of e-commerce on international trade in Asian countries. For this, dynamic panel data analysis is employed. Eq 1 is the expression of the dynamic panel data equation as follows.

$$Y_{it}=\beta_{0}+\beta_{1}Y_{it-1}+\beta_{2}X_{it}+\beta_{3}Z_{it}+\alpha_{i}+\varepsilon_{it}$$ (1)

Where *Y*_*it*_ and *X*_*it*_ represent dependent and independent variables. *Z*_*it*_ indicates control variables. The lag dependent variable is shown as *Y*_*it-1*_. Symbol *β* represents the estimated coefficient. Symbol *α*_*i*_ represents time-invariant unobserved heterogeneity. Symbol *ε*_*it*_ represents the idiosyncratic error term, whereas subscripts _i_ and _t_ represent country and time, respectively.

$${TRADE}_{it}=\beta_{0}+\beta_{1}{TRADE}_{it-1}{+\beta }_{2}{INTERNET}_{it}+\beta_{3}{TEL}_{it}+\beta_{4}{BROAD}_{it}+\beta_{5}{MOB}_{it}+\beta_{6}{SERVER}_{it}+\beta_{7}{GFCF}_{it}+\beta_{8}{INF}_{it}+\beta_{9}{GOV}_{it}+\beta_{10}{FDI}_{it}+\beta_{11}{EXG}_{it}+\beta_{12}{LAB}_{it}+\alpha_{i}+\varepsilon_{it}$$ (2)

Estimating static panel data analysis is not consistent due to endogeneity concern; hence this study employed the GMM estimator. Since this study contains a large number of cross sections and a few time periods, the GMM estimator of Arellano and Bover [29] and Blundell and Bond [30] is appropriate and were widely used widely in previous literature. Eq 2 represents the expanded form of the econometric model. Compared to the one-step GMM estimator, previous studies [16, 18, 27] show that the two-step GMM estimator is efficient. Therefore, this study follows the two-step system GMM.

The high correlation between independent variables may cause multicollinearity. However, highly correlated variables do not cause bias in the estimated coefficients results. Increasing the sample size is the best way to reduce multicollinearity [31, 32] issues. Accordingly, the present study covers several samples in the Asian continent, which address the concerns of multicollinearity and reliability of the findings.

### Variables and descriptive statistics

This study utilized a reliable dataset for 38 Asian nations, including those in the Middle East, the Far East, North and Central Asia, Southeast Asia, and South Asia. The sample country list along with data, is presented in S1 Data. The dataset for this study was obtained from the World Bank organization, covering eleven years from 2010 to 2020. Table 1 indicates definitions for variables in this study. Since there are no standard variables to represent e-commerce in secondary data, this study utilizes ICT variables because ICT infrastructure is essential to perform e-commerce. INTERNET, TEL, BROAD, MOB and SERVER are utilized as a proxy for e-commerce, whereas the dependent variable TRADE is used as a proxy for international trade along with the following control variables such as GFCF, INF, GOV, FDI, EXG and LAB.

**Table 1:** Sources for data collection.

Variable	Definition	Measurement
TRADE	Net trade in goods and services	Bop, current US$ (Billion)
INTERNET	Individual using the internet	% Of population
TEL	Fixed telephone subscribers	Per 100 people
BROAD	Fixed broadband Subscribers	Per 100 people
MOB	Mobile cellular subscribers	Per 100 people
SERVER	Secure internet servers	Per 1 million people
GFCF	Gross fixed capital formation	% Of GDP
INF	Inflation	Consumer prices annual %
GOV	General government final consumption expenditure	% Of GDP
FDI	Foreign direct investment, net inflows	% Of GDP
EXG	Official Exchange Rate	LCU per US$, period average
LAB	Labor force participation	% Of total population ages 15+ modeled ILO estimate

Regional-wise summary descriptive is presented in Table 2. All variables have the same number of observations which is 418. The highest average TRADE is USD 120 billion from North Asia. China had the highest TRADE at USD 370 billion in 2020 and India showed the lowest in 2012 with USD -136 billion. INTERNET is measured by the population percentage, with the highest number of users from West Asia at 99.70 percent and the lowest in Southeast Asia at 0.25 percent in Myanmar in 2010. TEL, BROAD, and MOB are the main independent variables of this study, measured by—per 100 people. East Asia has the highest TEL and BROAD. Western Asia has the highest MOB per 100 people at 210.0492 observed from Bahrain in 2016 whereas the lowest shows from Southeast Asia at 1.173894. SERVER users were observed to be on the lowest average USD 118.4653 million from South Asia. The highest average of GFCF is 33.93 percent from Central Asia. The lowest standard deviation of GFCF, with 0.48 percent is from North Asia. The INF is measured by consumer price annual percentage and shows both the largest and the lowest from Lebanon with percent of 84.87 and -3.75, respectively. The highest average GOV occurs at 18.38 percent in North Asia. Mongolia had the lowest FDI rate at -37.17 percent in 2016. The highest LAB has been noticed in Southeast Asia at 85.44 percent, followed closely by South Asia at 83.37 percent.

**Table 2:** Descriptive statistics of variables.

Asia	Variables												
		TRADE	INTERNET	TEL	BROAD	MOB	SERVER	GFCF	INF	GOV	FDI	EXG	LAB
North	Mean	120.00	69.272	24.901	18.004	155.643	3018.40	21.444	6.539	18.387	1.803	49.696	62.560
	Max	165.00	84.995	31.305	23.211	165.661	13344.76	21.982	15.534	20.706	3.019	72.105	62.860
	Min	66.30	43.000	18.971	10.942	141.787	17.09	20.612	2.878	17.626	0.503	29.382	61.890
	St.de	33.10	13.249	4.487	4.169	7.699	4538.81	0.480	3.529	0.857	0.966	17.286	0.284
Central	Mean	3.52	38.482	9.426	4.160	114.948	261.66	33.927	6.313	14.806	3.604	95.794	59.295
	Max	38.20	85.943	26.036	14.248	180.493	3307.64	69.673	16.636	20.912	17.131	412.953	70.890
	Min	-4.41	11.550	2.828	0.062	57.522	0.27	21.177	0.389	8.321	-5.190	4.379	40.468
	St.de	10.90	22.899	7.858	4.772	28.718	647.58	13.449	3.088	3.669	3.952	108.819	10.879
East	Mean	67.20	64.163	32.386	24.201	113.233	2127.65	31.996	3.064	16.271	4.116	805.014	63.148
	Max	370.00	96.505	60.250	43.555	152.032	22925.88	48.412	14.330	21.085	43.912	2813.290	71.070
	Min	-129.00	10.200	4.885	2.822	62.755	1.20	18.277	-0.742	12.255	-37.173	6.143	58.730
	St.de	114.00	28.469	20.878	13.126	19.549	4663.87	8.532	3.499	2.419	11.343	863.511	4.288
Western	Mean	4.25	60.479	16.444	12.276	115.528	702.44	22.199	4.612	16.345	3.385	262.624	55.591
	Max	18.40	99.701	47.197	30.063	210.049	12349.33	39.375	84.864	30.003	11.883	1507.500	72.276
	Min	-68.90	2.500	3.520	0.010	57.816	0.03	2.845	-3.749	7.661	-4.542	0.376	37.786
	St.de	34.50	22.429	9.872	7.464	30.287	1957.80	6.266	8.348	4.739	3.210	503.935	10.937
South	Mean	-21.40	20.935	4.441	2.875	98.562	118.47	27.064	6.219	10.432	2.647	84.469	58.206
	Max	0.55	63.186	17.690	11.782	181.329	1069.30	46.490	12.939	22.643	17.138	185.593	83.371
	Min	-136.00	3.700	0.443	0.228	34.041	0.20	12.521	-1.370	5.039	0.134	12.800	44.925
	St.de	30.00	16.227	4.580	2.698	35.329	194.37	7.325	3.211	3.982	3.887	43.273	11.448
Southeast	Mean	12.30	41.567	10.758	6.426	116.214	4182.44	27.854	3.249	17.470	5.889	4273.952	69.207
	Max	109.00	95.000	38.901	28.157	186.159	128377.70	70.105	18.678	103.173	29.690	23208.370	85.440
	Min	-39.40	0.250	0.153	0.003	1.174	0.02	15.918	-1.470	4.807	-1.321	1.000	54.750
	St.de	28.70	27.248	10.614	7.793	37.112	18472.62	7.333	3.170	17.054	6.245	6777.681	6.610

Source: Authors’ calculation based on data from the world bank.

Table 3 represents the correlations matrix of variables which was used to measure the effect of e-commerce on international trade in Asian countries. Correlation among the variables such as TRADE, INTERNET, TEL, BROAD, MOB, SERVER, GFCF, INF, GOV, FDI, EXG and LAB are examined in this study. The results of the correlation matrix show that BROAD was positively associated with TEL. The correlation between BROAD and TEL is 0.8367, indicating that users who subscribed to fixed broadband also probably subscribed to fixed telephone. Additionally, the correlation between BROAD and INTERNET is 0.7699. The correlation between MOB and SERVER is 0.1549, which indicates mobile cellular subscribers do not rely on secured internet servers in Asian countries. Even though there is a positive correlation among the variables, finding out whether the correlation is significant is vital. For this purpose, a significant test was performed and the p-values were observed.

**Table 3:** Correlation matrix.

Variable	TRADE	INTERNET	TEL	BROAD	MOB	SERVER	GFCF	INF	GOV	FDI	EXG	LAB
**TRADE**	1.0000											
**INTERNET**	0.2685***	1.0000										
**TEL**	0.2805***	0.6482***	1.0000									
**BROAD**	0.4009***	0.7699***	0.8367***	1.0000								
**MOB**	0.2569***	0.5318***	0.2797***	0.3798***	1.0000							
**SERVER**	0.1779***	0.2381***	0.2243***	0.2604***	0.1549***	1.0000						
**GFCF**	0.1417***	-0.1199**	-0.1140**	-0.0884	-0.0684	-0.0514	1.0000					
**INF**	-0.1448***	-0.2697***	-0.2071***	-0.2543***	-0.2533***	-0.1028**	-0.0425	1.0000				
**GOV**	0.0530	0.0550	0.0005	0.0270	-0.0200	-0.0292	0.3189***	-0.1044**	1.0000			
**FDI**	0.0736	0.0526	0.0747	0.0516	0.1481***	0.3432***	0.0968**	0.0602	-0.1 008**	1.0000		
**EXG**	-0.0443	-0.1134**	-0.1260***	-0.1231**	0.0345	-0.0404	0.0038	0.0236	-0.2167***	0.0488	1.0000	
**LAB**	0.2296***	0.0693	0.1152**	0.0891	0.2309***	0.0913	0.2458***	-0.1576***	-0.0021	0.2078***	0.3327***	1.0000

Note: ***, ** indicate significance at 1% and 5% levels, respectively.

To determine our variables at stationary, Levin–Lin–Chu test, Breitung test, Augmented Dickey Fuller test, Harris–Tzavalis test and Im–Pesaran–Shin test were used to test the existence of unit root in our dataset. Table 4 shows the results of stationary tests. The Levin–Lin–Chu test results indicate that the null hypothesis is rejected in the panel unit root test, except for TEL, BROAD and LAB. After changing the first difference, it becomes stationary. According to the results of the Breitung test, the null hypothesis is not rejected in the unit root test, except for TRADE, INF, and FDI. The Breitung test had the most non-stationary variables. After modifying variables into the first difference makes all variables stationary. Results of Augmented Dickey Fuller test null hypothesis is rejected, except for TRADE, SERVER, INF, GOV, and LAB. After taking the first difference, these variables turns stationary. The results of Harris–Tzavalis test does not reject the null hypothesis, except for TRADE, INF, and FDI. After taking the first difference, these non-stationary variables change to stationary. Im–Pesaran–Shin test results show the null hypothesis of unit root test is not rejected, except for MOB, FDI, and INF. After, changing the first difference, all non-stationary variables come to stationary level. Overall, taking/eliminating the first difference of non-stationary variables make all variables into stationary.

**Table 4:** Stationary test.

**Panel unit root test at level**					
**Variables**	**LLC test**	**Breitung test**	**ADF test**	**HT test**	**IPS test**
	**t-value**	**t-value**	**X2-value**	**z-value**	**W-t bar**
TRADE	-7.3568***	-2.2281**	70.7264	-7.5449***	-0.4305
INTERNET	-2.7988***	8.4352	188.8101***	3.7629	4.5832
TEL	-0.8389	5.0147	144.5763***	2.5184	2.9037
BROAD	5.2810	9.2600	145.1418***	3.6763	10.0924
MOB	-3.6431***	3.6015	193.0445***	0.2631	-2.1890**
SERVER	-1.8414**	10.5611	11.5828	9.7194	20.7920
GFCF	-6.4640***	0.1860	257.0250***	-0.8918	-1.5174*
INF	-31.4033***	-4.4776***	91.2964	-7.3014***	-1.9338**
GOV	-9.6592***	0.7572	84.1405	-1.3443*	2.1641
FDI	-4.4902***	-2.7276***	201.6361***	-8.9485***	-3.5824***
EXG	-10.5558***	5.0879	133.1491***	3.0302	-16871
LAB	1.6122	3.2366	55.1903	4.2693	7.1717
**Panel unit root test at first difference**					
**Variables**	**LLC test**	**Breitung test**	**ADF test**	**HT test**	**IPS test**
	**t-value**	**t-value**	**X2-value**	**z-value**	**W-t bar**
D.TRADE	-11.6420***	-5.0651***	314.9659***	-13.0706***	-5.6264***
D.INTERNET	-12.0179***	-3.9644***	196.0326***	-18.5237***	-3.6657***
D.TEL	-8.9415***	-3.0330***	397.1645***	-21.3033***	-5.7357***
D.BROAD	-1.8097**	-3.2274***	227.0422***	-14.3794***	-3.5623***
D.MOB	-8.9604***	-3.8651***	385.7016***	-14.9978***	-5.7779***
D.SERVER	4.9462	-3.2253***	101.1567**	-4.8632***	-0.1243
D.GFCF	-9.9382***	-3.7888***	341.1858***	-18.8385***	-5.6509***
D.INF	-14.1396***	-7.6257***	606.3573***	-23.7881***	-8.4688***
D.GOV	-4.7078***	-4.3384***	186.1239***	-19.5787***	-2.4325***
D.FDI	-8.5863***	-6.7565***	536.6869***	-23.6043***	-8.2746***
D.EXG	-45.9790***	-3.3220***	327.8905***	-13.8802***	-2.7546***
D.LAB	3.2005	-3.9918***	166.4353***	-15.2028***	-0.4546

Note: ***, **, * indicate significance at 1%, 5% and 10% levels, respectively.

Panel cointegration test indicates a stable relationship among the variables in the long term. The Kao cointegration test results showing the output of the five tests are depicted in Table 5. Estimated results indicate cointegration among the variables at 1% and 5% significance levels. A long-run relationship could be seen between each variable (INTERNET, TEL, BROAD, MOB, SERVER, GFCF, INF, GOV, FDI, EXG) and LAB due to the rejection of the null hypothesis at the 5% significance level. Since variables are cointegrated and stationary at the first difference, it is possible to conduct the analysis.

**Table 5:** Results of cointegration test.

Kao test for Cointegration		
	Statistic	p-value
Modified Dickey-Fuller t	-2.1385**	0.0162
Dickey-Fuller t	-3.1029***	0.0010
Augmented Dickey-fuller t	-8.7388***	0.0000
Unadjusted modified Dickey-fuller t	-4.5274***	0.0000
Unadjusted Dickey-fuller t	-4.3278***	0.0000

Note: ***, ** indicates significance at 1% and 5% levels, respectively.

Autocorrelation is a statistical measure that shows how closely related a data point is to the one preceding or following it in time. Heteroskedasticity refers to the variance of errors is not stable across the observations. In our study, the Breusch-pagan test and Breusch-Godfrey LM test are used to check the heteroskedasticity and autocorrelation correspondingly. Tables 6 and 7 show the results of both tests, respectively. The Breusch Godfrey test shows that the null hypothesis does not have a serial correlation between the variables, whereas the alternative hypothesis depicts a serial correlation between variables. The rejection of the null hypothesis at a 1% significance level indicates an autocorrelation among the residuals in the model.

**Table 6:** Results of autocorrelation test.

lags(*p*)	chi2	Degree of freedom	Prob > chi2
1	249.207***	1	0.0000

Note: *** indicates that significant at 1% level.

**Table 7:** Results of heteroskedasticity.

H0	chi2(1)	Prob > chi2
**Constant variance**	167.11***	0.0000

Note: *** indicates significance at 1% level.

The Breusch Pagan test shows that the null hypothesis is constant variance are all equal, which means homoscedasticity whereas the alternative hypothesis represents constant variance is multiplicative of many variables which is heteroskedasticity. The null hypothesis is rejected at a 1% significance level and heteroskedasticity is seen in the residuals of the model.

## Results

A two-step system GMM estimator is employed to examine the research objectives of this study as well as overcome the problem of endogeneity, heteroskedasticity and autocorrelation. Table 8 depicts the estimated results of the main regression and robustness check. Column 1 represents the main regression results, whereas columns 2 and 3 depict the results of the robustness check. Estimated results of GMM indicate a significant impact of e-commerce on international trade in Asian countries.

**Table 8:** Results of two-step system GMM estimator.

Variables	Main Regression	Robustness check	
	[1]	[2]	[3]
L.TRADE	0.6714***	0.6718***	0.6711***
	(0.0008)	(0.0008)	(0.0006)
INTERNET	0.0176***	0.0148***	-0.0715***
	(0.0063)	(0.0057)	(0.0094)
TEL	-1.4520***	-1.4090***	-1.3960***
	(0.0444)	(0.0338)	(0.0216)
BROAD	-0.5670***	-0.5370***	
	(0.0366)	(0.0259)	
MOB	0.0029		-0.0023
	(0.0035)		(0.0042)
SERVER	-0.00009***	-0.00008***	-0.00010***
	(0.00001)	(0.00001)	(0.00001)
GFCF	0.1096***	0.1000***	-0.0231
	(0.0353)	(0.0346)	(0.0345)
INF	0.2007***	0.2038	0.1928***
	(0.0180)	(0.0109)	(0.0259)
GOV	-2.9060***	-2.9000***	-2.7670***
	(0.0848)	(0.0822)	(0.1080)
FDI	-0.0995*	-0.1092*	0.0635
	(0.0566)	(0.0595)	(0.0870)
EXG	0.0103***	0.0106***	0.0128***
	(0.0006)	(0.0006)	(0.0007)
LAB	-0.0096	-0.0263	-0.1401***
	(0.0228)	(0.0261)	(0.0252)
Observation	380	380	380
AR (2) p-value	0.3576	0.3586	0.3195
Sargan test p-value	0.6328	0.9819	0.9929

Note: ***, * indicates significance at 1% and 10% levels, respectively. Parentheses represent the standard error. AR (2) represents Arellano and Bond test for serial correlation of order 2.

Based on estimated results, Arellano and Bond [33] test performed for serial correlation of order 2 in the residuals does not reject the null hypotheses, indicating no evidence of model misspecification. Further, Sargan test examines the overidentifying restriction, i.e. checks the validity of the instruments used in simultaneous equation models. The results of the Sargan test indicate that overidentifying restrictions are valid by not rejecting the null hypothesis. In the results of the main regression, the main independent variables show a significant impact on TRADE except for MOB. Among significant independent variables, the estimated coefficient of INTERNET shows a positive impact on TRADE at a 1% significance level; this indicates that a 1 percent increase in individuals internet usage per population increases net trade USD by 0.0176 billion. Further, estimated coefficients of TEL, BROAD and SERVER indicates significance at 1% level and shows negative impact towards TRADE.

Compared to other control variables, coefficients of GFCF, INF and EXG indicates significance at a 1% level and show a positive impact on TRADE. Estimated coefficients of GOV and FDI are significant at 1% and 10% levels. Although, coefficients of GOV and FDI show negative coefficients towards TRADE. LAB shows negative coefficient towards TRADE but is not statistically significant. Robustness check ensures the reliability of our findings. To stimulate the empirical results of main regression, MOB and BROAD are selected for the robustness test. Columns 2 and 3 show the results of the GMM estimator after dropping MOB and BROAD, respectively. Further, the robustness check findings show no significant changes/variations between the estimated results of the main regression and robustness check models. In addition, sensitivity analysis is performed to examine the multicollinearity concerns among the independent variables. S1 Appendix shows results of sensitivity analysis and confirms that retaining ICT variables, FDI and EXG does not lead to any misspecification. Despite the correlation matrix, S2 Appendix represents the results of variance inflation factor, which indicates moderately correlated variables.

## Discussion

Rapid technological advancement and digitalization are influential and key players in diverse ways in the growth of the world’s economies. International trade has become much more digitalized due to modern technology and therefore has increasingly benefited from e-commerce. Infrastructure and e-commerce promotion strategies adopted vary depending on each continent, due to unique regional factors/ country-specific factors and economic policies. Our research demonstrates that e-commerce has a substantial influence on the international trade of Asian countries. In doing so, we contribute to bridging the existing empirical gap because a few studies have focused on the Asian continent. Our findings especially point out that variables, such as the INTERNET and SERVER play a significant role in promoting international trade among Asian nations. Affirming this, Ismail and Mahyideen [34] confirmed that individuals using the internet and secured internet servers/ SERVER positively affect imports and exports of Asian countries. Similar studies [26, 35–37] support the findings that e-commerce and ICT infrastructure significantly impact economic growth and international trade in Asian countries.

Moreover, a study by Hussain et al. [38] proved that INTERNET has a noticeable impact on the economic growth of South Asian countries compared to MOB and TEL. Since, a larger portion of/ the majority of Asian countries is categorized as middle-income countries, Farhadi et al. [39] indicated that specific policies should be implemented to raise the effect of ICT towards economic growth. Ismail [40] revealed that a high-speed connection for internet users effectively facilitates bilateral trade between Asian countries. Secured internet servers, i.e. SERVER are essential components for ICT infrastructure. Due to data unavailability, most past literature does not include secured internet servers/ SERVER to measure the effectiveness of ICT indicators in economic growth. Thus, this study contributes to the existing literature.

This study utilizes the variable SERVER to address the previous research gaps. However, our study findings highlight that TEL and BROAD show negative coefficients towards international trade, in contrast to similar studies. In Asia, only a few countries, such as China, India, Malaysia, United Arab Emirates and Singapore have advanced in e-commerce than others. Most Asian countries can be suggested to capitalize on the right mix of ICT skills and facilities aligning with their global trade policies as prerequisites to reap the benefits of e-commerce to the fullest potential. Our study suggests implementing ICT infrastructure, providing ICT-related literature and technological investments via e-commerce can enhance trade in Asia. This study addresses the lacuna in the existing literature due to limited studies on e-commerce and international trade in the Asian continent and on the influential factors. Consequently, it provides insights to key stakeholders for effective decision making to boost trade via e-commerce in Asia. A key shortcoming of this research is the limited scope of the study, having analyzed 38 Asian countries due to limited data available on Asian countries.

## Conclusion and policy implication

This research clarifies the connection between global trade and e-commerce in Asian nations and its importance. The present study contributes to the literature by addressing the research gap based on the relationship between e-commerce and international trade, which has been found in previous literature. E-commerce has the potential to aid in the extensive growth of Asian countries. Therefore, key stakeholders/key actors should use these findings to devise policies/regulations to stimulate international trade, thereby aiming for economic growth.

Independent variables INTERNET, TEL, BROAD, MOB and SERVER are utilized as proxies for e-commerce; the dependent variable TRADE is used as a proxy for international trade along with following control variables such as GFCF, INF, GOV, FDI, EXG and LAB from 2010 to 2020. This study demonstrated that several factors had a positive influence on international trade between Asian nations, which offered support for the research objective that had been prior developed.

The estimated results of the two-step system GMM indicate a significant impact of e-commerce on the international trade of Asian nations. Furthermore, the results revealed that the main independent variables significantly impact TRADE except for MOB. Conversely, the other significant independent variables reveal a negative influence on TRADE; the estimated coefficient of the INTERNET shows a positive impact on TRADE. Moreover, GFCF, INF, and EXG are significant and positively impact TRADE compared to other control variables. Therefore, the findings of the study indicate that e-commerce influences international trade among Asian nations.

This study contains a few limitations which can be addressed in future research. Ten countries were eliminated from the study due to the unavailability of data. Due to this reason, the time span of this study was limited to 11 years from 2010–2020, especially due to a lack of certain ICT variable such as SERVER. As such, the years 2021 and 2022 were not examined/covered in this study. The findings of the empirical study stress that the governments of Asian nations should emphasise economic planning to boost e-commerce indicators to achieve the targeted goals in international trade.

Following are some potential policy ramifications of the research findings, which can be valuable to potential stakeholders to make trustworthy investment choices as knowledgeable and responsible investors.

Furthermore, governments of Asian countries should improve e-commerce regulation by establishing clear and consistent legislative regulations that encourage healthy competition and correct for any flaws in the current market. In addition, implementing an appropriate e-government system may help raise awareness about the benefits of good governance and boost the effectiveness of government operations. E-commerce in the private sector can be encouraged/incentivized via a multitude/ a variety of policy approaches and adjustments to policy instruments, including the elimination of taxes/relaxing taxes and the creation of public-private partnerships for the improvement of telecommunications infrastructure and e-commerce.

## Supporting information

S1 Data (XLSX)

S1 Appendix Sensitivity analysis. (DOCX)

S2 Appendix Variance inflation factor. (DOCX)
