# Supplementary material for: Does e-commerce really matter on international trade of Asian countries: Evidence from panel data
Source: PLoS One. 2023 Apr 24;18(4):e0284503. doi: 10.1371/journal.pone.0284503 (PMC10124838; doi:10.1371/journal.pone.0284503)
Supplement: S1 Appendix — (DOCX) [file pone.0284503.s002.docx]

**S2 Appendix. Sensitivity Analysis.**

| **Dependent variable** | **TRADE** | | | | | | | |
| --- | --- | --- | --- | --- | --- | --- | --- | --- |
| **Dropping variables** | **BROAD** | | **TEL** | | **GFCF** | | **EXG** | |
| **Variables** | **REM** | **FEM** | **REM** | **FEM** | **REM** | **FEM** | **REM** | **FEM** |
| INTERNET | -0.1476 | -0.2319 | -0.1485* | -0.1557* | -0.1122 | -0.1285 | -0.1429 | -0.1482* |
|  | (0.1641) | (0.1601) | (0.0886) | (0.0912) | (0.0849) | (0.0896) | (0.0870) | (0.0814) |
| TEL | 0.4905 | 0.1876 |  |  | 0.5815 | 0.1286 | 0.5628 | 0.0267 |
|  | (0.3361) | (0.3906) |  |  | (0.3552) | (0.3211) | (0.3459) | (0.2986) |
| BROAD |  |  | -0.0023 | -0.5378 | 0.0003 | -0.5411 | 0.0586 | -0.5356 |
|  |  |  | (0.6440) | (0.6985) | (0.6521) | (0.6726) | (0.6484) | (0.6804) |
| MOB | 0.1659 | 0.1227 | 0.1672 | 0.1303 | 0.1579 | 0.1094 | 0.1704 | 0.1329 |
|  | (0.1102) | (0.0917) | (0.1183) | (0.0971) | (0.1184) | (0.0960) | (0.1227) | (0.0982) |
| SERVER | 0.0004*** | 0.0004*** | 0.0003*** | 0.0003*** | 0.0004*** | 0.0004*** | 0.0004*** | 0.0004*** |
|  | (0.0001) | (0.0001) | (0.0001) | (0.0001) | (0.0001) | (0.0001) | (0.0001) | (0.0001) |
| GFCF | -0.4058* | -0.4511** | -0.4342* | -0.4464** |  |  | -0.3764 | -0.4494** |
|  | (0.2180) | (0.1831) | (0.2302) | (0.1898) |  |  | (0.2333) | (0.1907) |
| INF | -0.0347 | -0.0525 | -0.0557 | -0.0770 | 0.0038 | -0.0299 | -0.0287 | -0.0777 |
|  | (0.1360) | (0.1414) | (0.1290) | (0.1413) | (0.1346) | (0.1317) | (0.1319) | (0.1377) |
| GOV | -0.1727 | -0.3060 | -0.1553 | -0.2398 | -0.3572 | -0.5152 | -0.1494 | -0.2324 |
|  | (0.4705) | (0.6090) | (0.4182) | (0.5538) | (0.4438) | (0.5903) | (0.4143) | (0.5536) |
| FDI | 0.1108 | 0.1679 | 0.1346 | 0.1611 | -0.0494 | -0.0113 | 0.0950 | 0.1582 |
|  | (0.1334) | (0.1145) | (0.1456) | (0.1220) | (0.0967) | (0.1019) | (0.1434) | (0.1233) |
| EXG | -0.0002 | 0.0012 | -0.0008 | 0.0007 | -0.0002 | 0.0012 |  |  |
|  | (0.0009) | (0.0024) | (0.0009) | (0.0023) | (0.0008) | (0.0026) |  |  |
| LAB | 0.1307 | -0.9744 | 0.3429 | -0.8954 | 0.1660 | -1.1060 | 0.2625 | -0.8883 |
|  | (0.6285) | (1.0860) | (0.5237) | (0.9976) | (0.5833) | (1.0450) | (0.5070) | (1.0060) |
| Constant | -1.8520 | 80.300 | -6.6190 | 78.490 | -13.530 | 82.800 | -13.620 | 78.010 |
|  | (37.810) | (67.330) | (32.940) | (65.570) | (32.400) | (65.640) | (30.330) | (64.670) |
| R-Squared-within | 0.0397 | 0.0479 | 0.0386 | 0.0504 | 0.0308 | 0.0450 | 0.0367 | 0.0503 |
| between | 0.0681 | 0.0751 | 0.0228 | 0.1848 | 0.1251 | 0.1457 | 0.1001 | 0.1690 |
| overall | 0.0621 | 0.0375 | 0.0256 | 0.1051 | 0.1046 | 0.0862 | 0.0873 | 0.0939 |

**Table S1. Estimated results of fixed effect and random effect after dropping multicollinearity variables.**

Note: ***, ** and * indicates that significant at 1%, 5% and 10% level, respectively. Parentheses represent the robust standard error. FEM and REM represent the Fixed Effect Model and Random Effect Model, respectively.

| **Dependent variable** | **TRADE** | | |
| --- | --- | --- | --- |
| **Dropping variables** | **TEL** | **GFCF** | **EXG** |
| **Variables** | **Two-step GMM** | **Two-step GMM** | **Two-step GMM** |
| L.TRADE | 0.6740*** | 0.6709*** | 0.6561*** |
|  | (0.0010) | (0.0008) | (0.0011) |
| INTERNET | -0.0512*** | 0.0001 | 0.1355*** |
|  | (0.0116) | (0.0058) | (0.0056) |
| TEL |  | -1.4800*** | -1.6310*** |
|  |  | (0.0394) | (0.0377) |
| BROAD | -0.5635*** | -0.5277*** | -0.8475*** |
|  | (0.0155) | (0.0290) | (0.0439) |
| MOB | 0.0494*** | 0.0014 | 0.0324 |
|  | (0.0034) | (0.0029) | (0.0038) |
| SERVER | 0.000002 | -0.00009*** | -0.00010*** |
|  | (0.000008) | (0.00001) | (0.00001) |
| GFCF | 0.3136*** |  | 0.1159*** |
|  | (0.0373) |  | (0.0282) |
| INF | 0.1996*** | 0.1900*** | 0.1332*** |
|  | (0.0260) | (0.0161) | (0.0369) |
| GOV | -2.9280*** | -2.8470*** | -3.0710*** |
|  | (0.0751) | (0.0492) | (0.0702) |
| FDI | -0.1375*** | -0.0429 | -0.1210* |
|  | (0.0280) | (0.0409) | (0.0508) |
| EXG | 0.0151*** | 0.0098*** |  |
|  | (0.0007) | (0.0007) |  |
| LAB | -0.7213*** | 0.0417* | 0.0597 |
|  | (0.0114) | (0.0229) | (0.0400) |
| Observation | 380 | 380 | 380 |
| AR (2) p-value | 0.3420 | 0.3525 | 0.3422 |
| Sargan test p-value | 0.9963 | 0.6105 | 0.9993 |

**Table S2. Estimated results of two-step system generalized method of moments estimator after dropping multicollinearity variables.**

Note: ***, * indicates that significant at 1% and 10% level, respectively. Parentheses represent the standard errors. GMM depicts Generalized Method of Moments.
