# Supplementary material for: Does e-commerce really matter on international trade of Asian countries: Evidence from panel data
Source: PLoS One. 2023 Apr 24;18(4):e0284503. doi: 10.1371/journal.pone.0284503 (PMC10124838; doi:10.1371/journal.pone.0284503)
Supplement: S2 Appendix — (DOCX) [file pone.0284503.s003.docx]

**S3 Appendix. Variance inflation factor.**

| **Variable** | **VIF** | **1/VIF** |
| --- | --- | --- |
| BROAD | 4.84 | 0.206746 |
| TEL | 3.47 | 0.288293 |
| INTERNET | 3.03 | 0.330326 |
| MOB | 1.55 | 0.643750 |
| LAB | 1.38 | 0.724519 |
| GFCF | 1.27 | 0.788843 |
| FDI | 1.25 | 0.803105 |
| EXG | 1.23 | 0.810787 |
| SERVER | 1.23 | 0.811348 |
| GOV | 1.21 | 0.823640 |
| INF | 1.15 | 0.865897 |
| Mean VIF | 1.96 |  |

**Table S3. Estimated results of variance inflation factor.**
